# Circular RNAs as Novel Regulators of β-Cell Functions under Physiological and Pathological Conditions

**DOI:** 10.3390/ijms22041503

**Published:** 2021-02-03

**Authors:** Flora Brozzi, Romano Regazzi

**Affiliations:** 1 Department of Fundamental Neurosciences, University of Lausanne, 1005 Lausanne, Switzerland; Flora.Brozzi@unil.ch; 2 Department of Biomedical Sciences, University of Lausanne, 1005 Lausanne, Switzerland

**Keywords:** diabetes, pancreatic islet, insulin, non-coding RNA

## Abstract

Circular RNAs (circRNAs) constitute a large class of non-coding RNAs characterized by a covalently closed circular structure. They originate during mRNA maturation through a modification of the splicing process and, according to the included sequences, are classified as Exonic, Intronic, or Exonic-Intronic. CircRNAs can act by sequestering microRNAs, by regulating the activity of specific proteins, and/or by being translated in functional peptides. There is emerging evidence indicating that dysregulation of circRNA expression is associated with pathological conditions, including cancer, neurological disorders, cardiovascular diseases, and diabetes. The aim of this review is to provide a comprehensive and updated view of the most abundant circRNAs expressed in pancreatic islet cells, some of which originating from key genes controlling the differentiation and the activity of insulin-secreting cells or from diabetes susceptibility genes. We will particularly focus on the role of a group of circRNAs that contribute to the regulation of β-cell functions and that display altered expression in the islets of rodent diabetes models and of type 2 diabetic patients. We will also provide an outlook of the unanswered questions regarding circRNA biology and discuss the potential role of circRNAs as biomarkers for β-cell demise and diabetes development.

## 1. Introduction

Circular RNAs (CircRNAs) are non-coding RNAs that form covalently closed circles. They are generally generated by covalent binding of the 5′ site of an upstream exon with the 3′ end of the same or of a downstream exon. CircRNAs are neither capped nor polyadenylated (in contrast to linear transcripts) and are resistant to exoribonuclease degradation. Therefore, the life span of circRNAs is usually longer (19–48 h) than that of linear transcripts (4–9 h). Once produced, circRNAs can remain in the nucleus or be exported to the cytoplasm, where they can accomplish different functions [1,2,3].

CircRNAs were initially identified in 1976 in plant viroids [4] and later in archaea [5], and animals [6]. Following high-throughput sequencing and bioinformatics analysis, thousands of circRNAs have been identified so far, and over 100,000 circRNAs are estimated to be expressed in human cells. Some of them are conserved between species (in particular between humans and mice), are tissue-specific, and modulate mRNA transcription, splicing, and translation, affecting diverse cellular processes [1,2,3]. Abnormal expression of circRNAs has been implicated in a wide range of human cancers [7], in metabolic dysfunction [8,9], and in cardiovascular diseases [10,11].

The discovery and characterization of circRNAs have revolutionized the RNA world and rewritten the relationship between different RNA species. Here we will illustrate the peculiar mechanisms that lead to their generation, their proposed biological roles, and their emerging contribution to the regulation of pancreatic β-cell function and dysfunction.

## 2. Biogenesis, Classification, and Degradation of circRNAs

**Figure 1:**
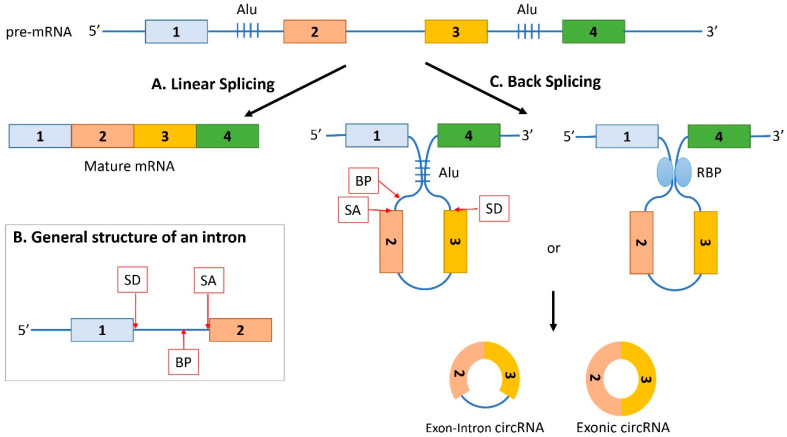
Generation of circRNAs by back splicing. (**A**) The immature pre-mRNA undergoes linear splicing where introns are removed, and exons are linearly joined to form the mature mRNA. (**B**) Introns contain different important sites for the splicing reaction: The splice-donor site (SD) at the 5’ left end, the splice-acceptor site (SA) at the 3’ right end, and the branch point (BP). (**C**) The immature pre-mRNA undergoes back splicing when there is a formation of a loop between the intron sequences flanking the downstream SD site and the upstream SA site. The formation of the loop is promoted by base pairing between inverted repeat elements of the flanking introns, such as Alu elements (Alu) or by the dimerization of RNA-binding proteins (RBP) that specifically bind to the flanking introns. Consequently, an upstream BP extends to a downstream SD site and allows the formation of a covalent binding between the 5′ site of an upstream exon and the 3′ end of a downstream exon, creating an Exon-Intron circRNA. If the intron is spliced-out from a transient Exon-Intron circRNA, or the covalent binding occurs between the 5′ site of an exon with the 3′ of the same exon, an Exonic circRNA originates.

According to the genomic sequences from which they originate, circRNAs are classified as: Exonic, exonic-intronic, or intronic. To understand how circRNAs are generated and then classified, it is fundamental to understand the splicing mechanisms. Linear splicing is defined as a process of mRNA maturation, where introns are removed from a precursor RNA and exons are linearly joined to form the mature mRNA (Figure 1A). Different sequences in the introns are important for the splicing reaction [12]: The splice-donor site (SD) at the beginning of an intron (5’ left end), the splice-acceptor site (SA) at the end of an intron (3’ right end), and the branch point (BP) (Figure 1B). The branch point is a sequence located anywhere from 18 to 40 nucleotides from the 3′ end of the intron. Successful splicing is assured by the specific interaction between different small nuclear ribonucleoproteins (snRNPs) of the spliceosome, and the above described intronic sequences [12].

The generation of circRNAs occurs through a process called back splicing [13]. Contrary to the canonical linear splicing, back splicing occurs after the formation of a loop between the intron sequences flanking the downstream SD site and the upstream SA site that brings them into proximity (Figure 1C). Consequently, an upstream BP extends to a downstream SD site and allows the physical interaction between the SA and the SD, and the covalent binding of the 5′ site of an upstream exon with the 3′ end of a downstream exon. The Exon-Intron circRNA (EI-circRNA) is then formed. If the intron is spliced-out from a transient EI circRNA, or the covalent binding occurs between the 5′ site of an exon with the 3′ of the same exon, an Exonic circRNA (E-circRNA) originates (Figure 1C).

The switch between linear splicing and back splicing is regulated by several factors. The looping of the introns (occurring in the back splicing) can be mediated by base pairing between inverted repeat elements (such as Alu elements), which are located in the upstream and downstream introns [14,15,16] or by the dimerization of RNA-binding proteins (RBPs) that bind to specific motifs in the flanking introns [17,18] (Figure 1C).

On the other hand, linear splicing is favored by exons surrounded by short flanking introns and/or by introns bound to the trans-acting RBPs double-stranded RNA-specific adenosine deaminase (ADAR1) and ATP-dependent RNA helicase A (DHX9). These RBPs disrupt base-pairing between inverted repeat elements and therefore prevent the looping of intron sequences, promoting canonical linear splicing [19,20,21]. ADAR1 is a highly conserved RNA-editing enzyme that binds double-stranded RNA and deaminates adenosine bases to inosine (A-to-I). Both ADAR1 and DHX9 bind to double-strand RNA formed by base pairing Alu elements and interfere with their stability. Indeed, a robust increase in circRNA expression was observed after depletion of both ADAR1 [16] and DHX9 [19]. ADAR1 downregulation also decreased hyper-editing events, suggesting a negative effect of A-to-I editing events on circRNA biogenesis [16]. Moreover, DHX9 depletion reduced translation of mRNAs containing inverted-repeat Alu elements in their 3′ UTRs, and this effect was rescued by overexpression of wild type DHX9, but not by a helicase-dead mutant. Therefore, the helicase activity of DHX9 seems to be crucial to resolve the double-stranded RNA structures originated by inverted Alu repeats and for the inhibition of circRNA synthesis [19].

**Figure 2:**
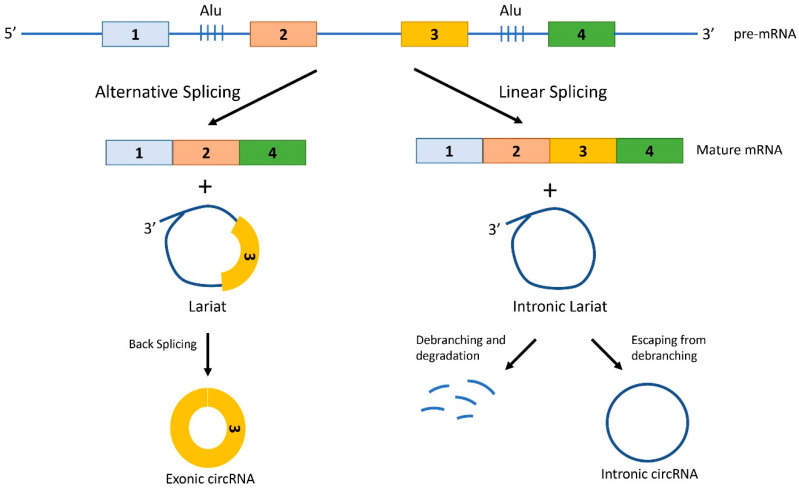
Generation of circRNAs from lariats. Lariats are lasso-shaped molecules that originate from pre-mRNAs during both alternative splicing and linear splicing. Lariats are formed when a 5′ end of a spliced intron is joined with a covalent bond to the BP adenosine of the intron. The lariat can either be linearized by specific RNA lariat debranching enzymes and thus be degraded, or escape debranching and form a stable circular transcript upon trimming of the 3′ linear tail. When lariats originate from linear splicing, they only contain introns and if they escape debranching, they produce Intronic circRNAs. However, lariats can also originate from alternative splicing, and therefore contain alternative exons together with the introns. Back splicing of hybrid exon-intron lariats generates Exonic circRNA.

Besides the back-splicing mechanism, circRNAs can originate also from the processing of lariat sequences [13,22]. Lariats are lasso-shaped molecules that originate from pre-mRNAs when a 5′ end of a spliced intron is joined to the BP adenosine of the intron with a 2′–5′ phosphodiester bond. These branched circRNAs containing a linear 3′ tail can be debranched (i.e., linearized) and degraded, or evade debranching and lose their 3′ tail, thus becoming stable circular transcripts. When lariats originate from linear splicing, they only contain introns (intronic lariat) and if they escape the debranching, they produce Intronic circRNAs (I-circRNAs) (Figure 2). However, lariats can also originate from alternative splicing, and therefore contain alternative exons together with the introns. Back splicing of hybrid exon-intron lariats generates E-circRNAs [13,22] (Figure 2)**.**

Following biogenesis, except for the intronic ones, most circRNAs, are exported to the cytoplasm [23], where they accomplish their functions, before degradation. Due to their closed circular structure, they are relatively stable molecules and their turnover implies mechanisms that differ from those of linear RNAs [2]. Several pathways have been proposed to contribute to circRNA degradation, but the precise mechanisms involved are still poorly understood. The depletion of GW182, a key component of processing bodies (P-bodies), has been reported to trigger the accumulation of cytoplasmic circRNAs in *Drosophila* S2 cells, and its overexpression decreased the levels of steady-state circRNAs [24]. Though, the molecular events involved in GW182 regulation of circRNA turnover are still unknown [24]. A different mechanism involving m6A methylation of circRNAs has also been described [25]. Indeed, upon m6A-modifications, circRNAs are recognized by YTHDF2, allowing this protein to form a complex with the adaptor protein HRSP12 and RNase P/MRP (endoribonucleases), that degrades the circRNAs [25]. Interestingly, m6A methylation of nascent pre-RNA favors the binding of the m6A reader protein hnRNPG, promoting alternative splicing vs. linear splicing [26], therefore indirectly regulating the production of the circRNAs. Finally, an Argonate2 (Ago2)-dependent cleavage has been reported for the exonic circRNA ciRS-7/Cdr1as. This circRNA contains a sequence with high complementarity to miR-671 that, upon binding of the microRNA (miRNA) (see Section 3.1), promotes the cleavage by Ago2 [27].

## 3. Biological Functions of circRNAs

The function of the majority of the circRNAs is unknown, but emerging evidence shows that they play important roles in many biological processes. Different types of circRNAs have distinct localizations, and consequently diverse functions. I-circRNAs and EI-circRNAs are found mainly in the nucleus, whereas the vast majority of the E-circRNAs are enriched in the cytoplasmic fraction [28]. Active transport processes of circRNAs from the nucleus to the cytoplasm have been described [23].

CircRNAs localized in the nucleus regulate gene expression through the modulation of transcription and/or alternative splicing [29]. Cytoplasmic circRNAs have diverse functions: They can act by sequestering miRNAs [30,31,32,33] or proteins [34,35], they can enhance protein activity [36,37], form scaffolds to mediate complex formation between specific enzymes and substrates [38,39], or recruit proteins to specific locations [40]. Furthermore, a subset of circRNAs undergo cap-independent translation under specific conditions [41,42,43,44].

### 3.1. circRNAs Acting as miRNA Sponges

MicroRNAs (miRNAs) are small non-coding RNAs that fine tune gene expression at the posttranscriptional level, by binding to the 3′ untranslated regions of target mRNAs and inhibiting their expression [45]. Cytoplasmic circRNAs can contain miRNA binding sites in their sequences and therefore sequester these small RNAs, preventing the interaction with specific mRNA targets. This way, circRNAs indirectly modulate the expression of the mRNA targeted by the sequestered miRNAs. In this scenario, it is important to consider the stoichiometric relationship between the miRNA binding sites present in the circRNA and the number of sites within the targets, as highly abundant circRNAs containing many binding sequences are more likely to compete with endogenous RNAs [31,46]. For example, the well-characterized circRNA, ciRS-7/Cdr1as, contains more than 70 conserved binding sites for miR-7 and is highly expressed in brain and pancreatic islets. Thus, this circRNA has the potential to regulate the expression of miR-7 target genes [30,32,33]. However, whether ciRS-7 inhibits or protects miR-7 from degradation may depend on the cellular context [32,47,48]. Indeed, removing the ciRS-7 locus from the mouse genome led to the reduction of miR-7 levels [32], whereas other studies found a negative correlation between ciRS-7 expression and miR-7 expression [47,48].

Of note, some circRNAs possess binding sites for many miRNAs rather than multiple sites for one particular miRNA. This is the case for the oncogenic circCCDC66, that contains binding motifs for several miRNAs, including miR-33b and miR-93, which target the MYC oncogene [49]. Consequently, some circRNAs can have either tumor-suppressive or oncogenic activities.

Many other circRNAs with miRNA sponging ability have been described, including circHIPK3 and circBIRC6 [50,51], and are reviewed in [14,31,50,52].

### 3.2. circRNAs Interacting with Proteins

One of the first circRNAs proposed to function as a protein sponge is circMbl, which originates from the gene that encodes the splicing factor muscleblind (mbl) in *D. melanogaster* and the homologous gene muscleblind-like protein 1 in humans (MBNL1). Interestingly, circMbl contains a binding site for mbl and MBNL1, respectively, and the introns flanking circMbl have many mbl (or MBNL1) binding sites [35]. The binding of mbl (or MBNL1) facilitates the looping of the nascent RNA to promote circMbl biogenesis. Therefore, an autoregulatory circuit may exist in which excess mbl or MBNL1 decreases the production of its own mRNA by promoting circRNA biogenesis, and the circRNA promotes the linear splicing of the gene by tethering mbl or MBNL1 [35].

Other circRNAs, such as circPABPN1 and circANRIL, also function by interacting with specific proteins [34,53]. circPABPN1 sequesters the protein RBP Hu-antigen R (HUR) and consequently suppresses the translation of nuclear poly(A) binding protein 1 (PABPN1) mRNA [34]. circANRIL impairs ribosome biogenesis by binding to pescadillo homologue 1 (PES1), an essential pre-ribosomal assembly factor, and consequently induces nucleolar stress and p53 activation [53].

Some others circRNAs, such as circAmotl1 [39] and circFoxo3 [38], function as scaffolds to facilitate the colocalization of enzymes with their substrates. CircAmotl1 physically binds to both PDK1 and AKT1 to facilitate PDK1-dependent phosphorylation and nuclear translocation of AKT1 in murine cardiomyocytes, epithelial cells, and endothelial cells [39]. In vivo, circ-Amotl1 expression has a cardioprotective effect in a mouse model of cardiomyopathy [39]. On the other hand, circFoxo3 promotes the interaction between mouse double-minute 2 (MDM2) and p53 in cancer cells, and, therefore, the degradation of p53 [38]. In the non-tumoral cell line NIH3T3, circ-Foxo3 binds to the cell cycle regulators cyclin-dependent kinase 2 (CDK2) and cyclin-dependent kinase inhibitor 1 (p21) to generate a ternary complex inhibiting the binding between the two proteins and repressing cell cycle progression at the G1 stage [54]. Circ-Foxo3 can also interact with the anti-senescence proteins ID1 and E2F1 and the stress-associated proteins FAK and HIF1a, retaining these proteins in the cytoplasm and blocking their function, thereby promoting cardiac senescence [55]. However, more recently, a miRNA-sponging activity has been attributed to circFoxo3 in tumors [56,57], demonstrating a potential flexibility of circRNA functions, depending on the cellular context. This demonstrates that some circRNAs are able to bind to several RBPs and can function as messengers carrying spatial and temporal biological information in various tissues, developmental stages, and cellular conditions.

Recently, an extensive screening of circRNA-RBP interactions has been performed, with the aim of obtaining a broader picture of the circRNA-RBP interactome [58]. A comprehensive catalogue of circRNA-RBP interactions in HepG2 and K562 cells was generated, and several candidates have been characterized [58]. In particular, it was demonstrated that circCDYL associates to IGF2BP1 and IGF2BP2, and that this interaction modulates proliferation of bladder cancer cells. Indeed, the inhibition of circCDYL perturbs the expression of key cancer genes, and elevated levels of circCDYL are linked to the overall survival of bladder cancer patients [58].

This study corroborates the concept that cell-type-specific circRNA-RBP interactions can play key regulatory roles in tumorigenesis and, in general, in the regulation of different cellular functions.

### 3.3. circRNAs Translated to Proteins

Even if circRNAs do not contain 5′ caps and poly(A) tails, some of them can be translated in a cap-independent manner. This is possible in case of the presence of internal ribosome entry sites (IRESs) [59] or the incorporation of an m6A modification in the 5′ untranslated region (UTR) of the RNA [60,61]. In fact, when Wang et al. engineered an IRES in a circRNA, a protein corresponding to the circRNA was translated [62]. Another study found that m6A modification is abundant in many circRNAs, and that the presence of this methylation drives their translation in a manner similar to IRES [42]. In this study, the efficiency of translation was modulated by the degree of m6A in the transcript [42]. Interestingly, these two cap-independent translation mechanisms may not be independent. Indeed, high degree of m6A methylation was detected in the IRES-activated protein-coding circRNA circZNF609 in myoblasts, suggesting a possible connection between these two cap-independent translation processes [44]. In this particular study, the circRNA-derived peptides corresponded to truncated versions of the canonical proteins, lacking essential functional domains; therefore, these peptides were found to act as dominant-negative protein variants [44]. Of particular interest is the discovery in *Drosophila* of a circRNA generated from the sulfateless gene (circSfl), which is consistently upregulated, particularly in brain and muscle, of different long-lived insulin mutant flies [63]. CircSfl was found to be translated, and circSfl protein levels were increased in insulin mutant flies. Moreover, overexpression of the circSfl ORF from a linear transcript extended the lifespan of female flies, indicating that the protein encoded by circSfl is sufficient to increase *Drosophila* lifespan [63]. Despite these interesting and promising findings, for most of the circRNA-derived peptides identified so far, the function, if any, remains unknown. 

### 3.4. circRNAs Regulating Mitochondrial Functions

Eukaryotic cells contain many copies of mitochondrial DNA [64]. Moreover, mitochondrial DNA is translated in pre-mRNA that can be processed by splicing. Indeed, mtRNA introns contain canonical splice sites, and the majority of the ribonucleoproteins that are part of the nuclear spliceosome machinery are imported into mitochondrial compartments and bind to mtRNA transcripts. Together, these observations confirm the occurrence of splicing in mtRNAs and suggest that the nuclear spliceosome complex can mediate mtRNA splicing within the organelle. In addition, the presence of hundreds of backsplicing junction reads supports the existence of mitochondria-encoded circRNAs (MecciRNAs) in different vertebrate species [65]. Detailed studies on MecciND1 and MecciDN5 further suggest that they may interact with nuclear encoded proteins and promote their importing into mitochondria [65].

Recent work by Zhao and colleagues demonstrate that the mitochondria-located circRNA SCAR is dysregulated in the liver of patients with non-alcoholic fatty liver disease (NAFLD). SCAR directly interacts with ATP5B, a subunit of the mitochondrial ATP synthase, and turns off mitochondrial permeability transition pore (mPTP). Lipid overload inhibits SCAR expression in fibroblast, and consequently mPTP is no longer inhibited, causing a deleterious rise in ROS formation [66]. Using experiments carried out in vivo, the authors proved that the human circRNA SCAR can interact with mouse ATP5B and is able to alleviate high fat diet-induced cirrhosis in mice, providing evidence for a potential therapeutic strategy to prevent the development of this disease [66].

## 4. circRNAs in Pancreatic Islet Cells

Pancreatic β-cells are the only cells in our body able to produce and secrete insulin in response to glucose and, therefore, are central players in the control of blood glucose homeostasis. Loss or dysfunction of β-cells can lead to the release of insufficient insulin to cover the organism needs, promoting diabetes development. Different classes of non-coding RNAs are involved in the regulation of β-cell functions and in diabetes development, and there is now emerging evidence that circRNAs may be among them [9].

The first circRNA studied in pancreatic islet cells was ciRS-7/CDR1as, which acts as a sponge of miR-7, one of the most abundant miRNAs present in β-cells. This circRNA is abundant, largely cytoplasmic, and originates from the antisense of the Cerebellar Degeneration-Related protein 1 gene (CDR1) [67]. Since it contains about 70 conserved miR-7 binding sites, it is either termed Cdr1as (antisense) or ciRS-7 (Circular RNA Sponge for miR-7) [67]. Overexpression of ciRS-7 in murine islet cells was found to increase insulin content and secretion [68]. Moreover, Myrip and Pax6, regulating insulin granule secretion and insulin transcription, respectively, were identified as new targets of miR-7 and were modulated by the level of ciRS-7.

Global profiling of the circRNAs using a microarray approach led to the identification of thousands of circRNAs in human pancreatic islets, 497 of which were also conserved in mouse islets [33]. Three of the most abundant circRNAs were further investigated: CircAFF1, and circHIPK3, which originate from exonic sequences of the Aff1 and Hipk3 genes, respectively, and ciRS-7/Cdr1as [33].

The expression of each of these circRNAs in β-cell lines and primary islet cells was confirmed using divergent primers. Moreover, the modulation of their expression with siRNAs directed against the circularized junctions, or against sequences specific to the circRNAs in rodent islet cells, revealed that they contribute to the regulation of insulin secretion and/or to the control of the β-cell mass. Indeed, circAFF1 deficiency was found to enhance apoptosis, but had no effect on β-cell proliferation or in glucose-stimulated insulin secretion. Interestingly, circHIPK3 and ciRS-7/CDR1as were reduced in the islets of diabetic *db/db* mice, which lack the leptin receptor and are severely obese. Mimicking this decrease in the islets of wild type animals resulted in impaired insulin secretion, reduced β-cell proliferation, and survival [33].

Silencing of ciRS-7/CDR1as results not only in a decrease in insulin secretion [33,68], but also reduces prolactin-stimulated proliferation of primary rat β-cells and MIN6 cells, without affecting β-cell survival. These data suggest that alterations of ciRS-7 levels under diabetic conditions may contribute to β-cell dysfunction and to the loss in the functional β-cell mass.

Moreover, circHIPK3 was found to control insulin mRNA levels and insulin secretion in pancreatic β-cells [33]. Indeed, the knockdown of circHIPK3 in MIN6 reduced insulin promoter activity. Microarray analysis after circHIPK3 knockdown resulted in a downregulation of genes involved in insulin secretion, including Akt1, Slc2a2, and Mtpn [33]. Similar changes in gene expression upon circHIPK3 knockdown were also detected in rat and human islets, corroborating the role of this circRNA in the regulation of glucose-stimulated insulin secretion, insulin biosynthesis, proliferation, and apoptosis.

Mechanistic studies revealed that circHIPK3 is likely to act as a miRNA sponge. Indeed, silencing of circHIPK3 resulted in the up-regulation of a large number of genes that are enriched for putative targets of miR-124-3p, miR-338-3p, miR-29b-3p, and miR-30 [33]. Moreover, after circHIPK3 silencing there was a decrease in the activity of a luciferase construct containing the 3′ UTR of human MTPN, which is known to be controlled by miR-124-3p [69]. This result is consistent with a rise in the repressive activity of miR-124-3p after circHIPK3 knockdown. Together, these observations suggest that circHIPK3 modulates the activities of β-cells by sequestering a group of miRNAs that control the expression of key β-cell genes, such as *Slc2a2*, *Akt1*, and *Mtpn* [33].

More recently, other circRNAs dysregulated in the islets of diabetic *db/db* mice were identified by high-throughput RNA sequencing [70]. This study discovered hundreds of circRNAs that display expression changes in mouse pancreatic islets; one of them, circTulp4, is downregulated in the islets of diabetic models and in MIN6 cells under lipotoxic condition. CircTulp4 was found to regulate cell proliferation through the interaction with miR-7222-3p, which inhibits the expression of sterol O-acyltransferase 1 (SOAT1). The accumulation of soat1 activates cyclin D1 expression, thus promoting cell cycle progression. These findings indicate that circTulp4 regulates β-cell proliferation via a signaling cascade including miR-7222-3p/soat1/cyclin D1 [70].

The first study aiming to investigate the transcriptional landscape of circRNAs in different human islet cell types, compared published RNA-seq datasets of FACS-sorted human α-, β-, and exocrine cells [71]. This analysis showed that circRNAs are highly abundant in both α- and β-cells and identified 10,830 circRNAs expressed in human α-, β-, and exocrine cells. More specifically, 36 circRNAs candidates were differentially expressed, 22 upregulated and 14 downregulated, in β-cells compared to α-cells, and about 400 circRNAs were found to be generated particularly in one or in the other islet cell type. Of these, seven circRNAs were highly selective for α-cells and one of them for β-cells. The identification of cell-specific α- and β-cell circRNAs suggests they may play a role in regulating specific cellular function. However, their biological role in islet cells remains to be elucidated. A more recent study of the whole circRNA profile carried out in human islets of healthy donors and T2D patients, identified 2619 circRNAs expressed in islet donors [72]. Among them, 13 co-localized with the GWAS association signal for T2D. Despite the number of circRNAs identified in this study is lower than in the previous one (10,830 circRNAs in Kaur et al.), there was a considerable overlap between the two circRNA profiles, especially for the first top 100 abundantly expressed transcripts. Out of the five most abundant circRNAs present in the islet cells, CAMSAP1, CIRBP, RPH3AL, RHOBTB3, and ZKSCAN1, four of them were differentially expressed in the islets of T2D donors [72]. Moreover, the expression of two of them changed in palmitate-treated human EndoC-βH1 cells. Of note, the level of circCAMSAP1 in the peripheral blood of patients with T2D showed a negative association with the diabetes status and might have a future utility as a biomarker [72].

Of particular interest was the recent discovery of β-cell specific circRNAs derived from the insulin gene [73,74]. To obtain a comprehensive picture of all circRNAs present in islet cells, a two-algorithm computational approach was used to de novo annotate potential circular transcripts detectable in high-throughput RNA-sequencing data from mouse pancreatic islets [73,75]. This computational approach predicted the expression of 15,925 putative circRNAs, which included circRNAs generated from key β-cell genes such as Chga, Chgb, Gck, Glp1r, Pcsk1, Pcsk2, and Slc30a [73]. Interestingly, this strategy led also to the identification of three potential circRNAs generated from the second intron of the insulin pre-mRNA that were named circular intronic Ins2 (ci-Ins2). The presence of these circular transcript was confirmed by RT-qPCR using specific divergent primers in mouse, rat, and human islets. Gel electrophoresis revealed the amplification of two or more qPCR products in DNase-treated and reverse-transcribed islet RNA from each of the three species. Sequencing of these qPCR products indicated two common types of non-colinear junctions between species corresponding to the lariat or to the totality (full length) of the second intron of the insulin pre-mRNA [73].

While this study was ongoing, Das et al. independently analyzed the same RNA-sequencing data set [75], using two different algorithms (CIRCexplorer2 and CIRI2A) and identified a total of 67,146 and 28,903 circRNAs, respectively [74]. Interestingly, this group also identified circRNAs generated from the mouse preproinsulin 2 (Ins2) gene, out of which only two were generated from exon 2, while the others were derived from intron 2 [74]. These computational data were further verified by RT-qPCR with divergent primers placed on exon 2 and intron 2. This led to the detection of multiple alternative circular transcripts, but the presence of exonic circ-Ins2 was not confirmed in mouse islets and in the murine βTC6 beta cells [74]. Furthermore, in agreement with the findings obtained in our laboratory [73], RT-PCR analysis using the primer spanning the circRNA junction proved the existence of multiple circular intronic RNAs generated from intron 2 [74], potentially originating by the recognition of multiple branchpoints [76].

In our study, we also examined ci-Ins2 functions in pancreatic β-cells, focusing on the lariat-derived circRNA [73], since this class of circRNAs has been shown previously to play important regulatory roles in other cell types [36]. No essential role could be attributed to rat ci-Ins2 in β-cell survival and proliferation. However, deficiency of this lariat in rat and human islet cells resulted in reduced insulin release in response to nutrients (glucose) and/or membrane-depolarizing compounds (KCl). Transcriptomic analysis after ci-Ins2 knock down revealed changes in the expression of key genes involved in the secretory pathway of β-cells [73]. Indeed, silencing of rat ci-Ins2, or human ci-INS, was associated with a reduction in a remarkable number of mRNAs encoding for essential components of the secretory machinery of β-cells, including the voltage-dependent Ca^2+^ channel subunit Cacna1d and different targets and regulators of Rab3 GTPases. Moreover, ci-Ins2 silencing also reduced the Ca^2+^ peak and integrated Ca^2+^ load in rat islet cells challenged with stimulatory and membrane-depolarizing K^+^ concentrations, confirming the impairment on Ca^2+^ dynamic during insulin secretion. Most of these changes were consistent in both species and were reproduced upon silencing with different gapmeR sequences, confirming the specificity of the effect [73].

**Figure 3:**
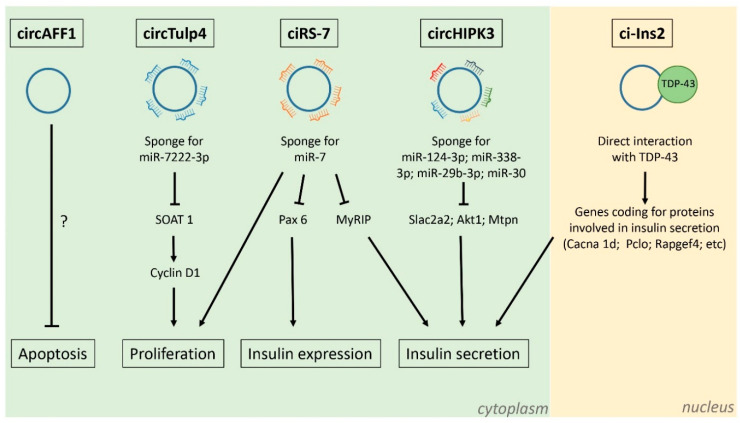
Functions of circRNAs expressed in pancreatic β-cells. CircTulp4 [70], circAFF1, ciRS-7, circHIPK3 [33], and ci-Ins2 [73] are among the most abundant circRNAs expressed in pancreatic β-cells. CircTulp4, ciRS-7, and circHIPK3 are exonic circRNAs mainly localized in the cytoplasm and act as microRNA sponges. They affect the expression of different proteins involved in β-cell proliferation, insulin biosynthesis, and insulin secretion. CircAFF1 is also exonic and inhibits β-cell apoptosis through an unknown mechanism. Ci-Ins2 is an intronic circRNA localized mainly in the nucleus and interacts with the RNA-binding protein TDP-43. Ci-Ins2/TDP-43 interaction promotes the expression of many genes coding for proteins involved in insulin secretion. CircTulp4, ciRS-7, circHIPK3, and ci-Ins2 expression is downregulated in islets from *db/db* mice. Moreover, the level of ci-Ins2 is also decreased in the islets obtained from T2D patients.

Computational analysis revealed that ci-Ins2 can potentially interact with several RBPs [73,74]. Indeed, the interaction with TARDNA-binding protein 43 kDa (TDP-43), an RBP identified as a potential ci-Ins2 target in both studies [73,74], was confirmed experimentally [73]. TDP43 is of particular interest because knockout of this RBP in β-cells results in defective insulin secretion [77]. Moreover, TDP-43 knockout and ci-Ins2 silencing result in a decrease in the expression of a common set of genes and, in particular, in genes involved in insulin exocytosis [77]. Taken together, our findings suggest that ci-Ins2 regulates insulin secretion in part through direct interaction with TDP-43 (Figure 3). It is plausible to hypothesize that ci-Ins2 functions as a co-activator of TDP-43 because the expression of most genes involved in insulin secretion is reduced upon silencing of TDP-43 or ci-Ins2.

Interestingly, the level of ci-Ins2 is reduced in the islets of rodent models of T2D as observed for circHIPK3 and ciRS-7 [33] and in palmitate and glucose treated βTC6 cells [74]. In addition, the level of ci-INS, the human homolog of ci-Ins2, is lower in the islets of T2D donors and is inversely correlated to HbA1c levels [73], suggesting that reduced ci-Ins2 and ci-INS may contribute to β-cell failure in T2D.

Taken together, these findings suggest that circRNAs may play a key role in the regulation of β-cell activity, and may be potentially involved in the pathogenesis of T1D and T2D. However, even if alteration in the expression of circRNAs have been demonstrated to play a role in the onset and/or progression of many autoimmune diseases [78,79], the potential contribution of circRNAs in T1D pathogenesis has been so far poorly investigated, especially in the β-cell context. Indeed, at present, the only evidence for a possible implication of circRNAs in T1D is the reduction in the expression of circARHGAP12 in the islets of pre-diabetic NOD mice, which spontaneously develop T1D [33]. However, the role, if any, of circARHGAP12 in β-cells remains to be clarified since silencing of this circRNA in MIN6B1 cells had no effect on proliferation, survival, or insulin secretion [33].

## 5. Circular RNAs as Potential Disease Biomarkers

Despite intensive research, the causes of pancreatic β-cell failure during the development of diabetes remain incompletely understood. The therapeutic strategies currently available to prevent the manifestation of the disease and delay its progression need to be ameliorated. The efficacy of the treatments would be greatly improved by implementing them during the initial phases of the disease and by identifying individuals with the highest risk of developing it. This goal can be achieved using biomarkers capable of predicting and/or monitoring the progression of T1D and T2D and their long-term complications. The level of circRNAs varies in different tissues and according to cellular conditions [7,80]. Moreover, as outlined in the previous chapters, the expression of some circRNAs is altered under disease states [7,8,9,10,11], including diabetes [72,73,81]. Finally, some circRNAs are secreted in the extracellular space and are abundant in body fluids (blood, saliva, urine, cerebrospinal fluid) and are detectable in liquid biopsies [82,83]. Thus, detailed analysis of the level of specific circRNAs may potentially be useful to monitor the functional state of β-cells under pre-diabetic conditions and to predict β-cell failure and demise.

Many circRNAs have been reported to be enriched in small extracellular vesicles called exosomes [84]. These vesicles are secreted by the cells in the extracellular space and in body fluids, and exosomal miRNAs are already used as biomarkers for different diseases [85,86]. There is emerging evidence indicating that the level of circRNAs in exosomes holds a prognostic and diagnostic potential. Thus, the level of *circNRIP1* in circulating exosomes is upregulated and correlates with tumor size in gastric cancer patients [87]. This circRNA acts as a sponge for miR-149-5p, affecting the expression level of *AKT1*. Consequently, *circNRIP1* promotes proliferation and migration in gastric cancer cells [87]. In another study, Xie et al. compared the circRNA expression profile in serum exosomes of colorectal cancer patients and healthy subjects, and found that exosomal circRNAs in cancer samples are more abundant than in control samples. Therefore, the analysis of exosomal circRNAs may provide novel powerful tools for early non-invasive diagnosis of cancer [88].

In view of the recent discovery of thousands of circRNAs in pancreatic islets and of the capacity of β-cells to release RNAs in different types of vesicles under physiopathological conditions [9], the analysis of circRNAs in body fluids may potentially yield precious information for identifying individuals at risk for developing diabetes or its long-term complications. The use of specific circRNAs as biomarkers for T1D and T2D occurrence and progression is attractive and deserves further investigation. However, several technical obstacles still need to be overcome. In fact, the analysis and the quantification of circRNAs is technically more complex than the measurement of other RNA classes such as miRNAs. The design of primers specifically amplifying a selected circRNA is often challenging [2]. This is usually achieved using convergent primers spanning over the circular junction that amplify a sequence differing from the corresponding linear transcript. However, this approach is often amplifying several circRNAs sharing the same circular junction, but including a different number of exons/introns. Another key issue, will be to identify the ideal candidate to mirror the functional state of the insulin-secreting cells. Ci-Ins2 would be a potentially attractive candidate since its expression is β-cell specific and decreases in the islets of T2D donors [73]. The first step would be to investigate if ci-Ins2 is detectable in human blood and whether its level changes in samples of T1D and T2D patients. In case of a lack of sensitivity due to the low number of copies present in the blood, it could be envisioned to assess whether ci-Ins-2 is present in exosomes or in other extracellular vesicles (EVs). Indeed, as mentioned above, some circRNAs are abundant in EVs circulating in the blood and other body fluids [84]. However, the exosomes released by β-cells represent only a very tiny proportion of the vesicles present in body fluids and routine isolation of EVs specifically released by β-cells is not yet possible. Thus, the analysis will need to be focused on circRNAs that are highly specific for β-cells to avoid confounding effects from vesicles released by other tissues. Microarray or RNA sequencing analysis may provide a broader picture of the circRNAs circulating in body fluids under healthy and diabetes conditions. Indeed, using a microarray approach, almost a hundred differentially expressed circular transcripts were identified in the blood of T1D patients, and the changes of six of them were confirmed by qRT-PCR [89]. Interestingly, one of them, Hsa_circ_0002473, originates from the DNAJC3 gene that also encodes for a GRP78-interacting protein that facilitates its membrane translocation in dying pancreatic β-cells [90]. Moreover, DNAJC3 loss-of-function mutations lead to a monogenic and recessive form of diabetes mellitus in humans. However, the tissue distribution of Hsa_circ_0002473 was not yet investigated and we still don’t know whether Hsa_circ_0002473 plasma levels correlate with β-cell-demise. Thus, further studies need to be performed to confirm whether Hsa_circ_0002473 represent a potential biomarker to monitor β-cell loss during T1D pathogenesis.

These technical obstacles are likely to be overcome in the near future thanks to the accumulating information about the expression and the role of circRNAs in β-cells. Solving these issues will be an essential prerequisite to open the way to a systematic assessment of the possible use of circulating circRNAs as T1D and T2D biomarkers.

## 6. Conclusions and Perspectives

**Figure 4:**
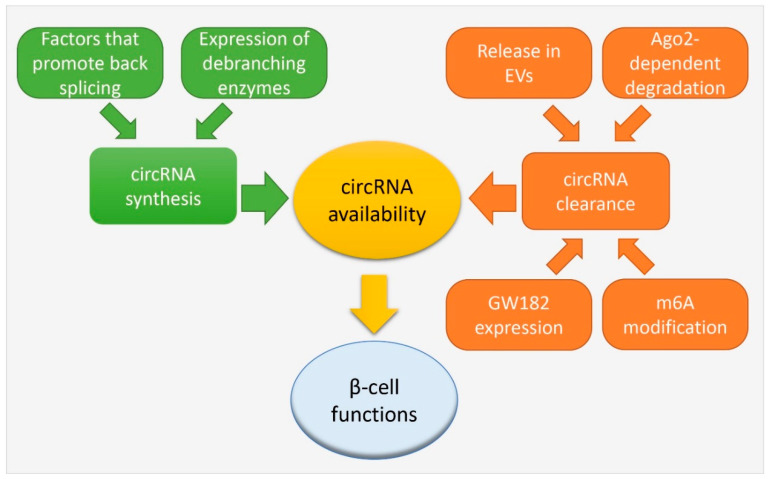
Known mechanisms regulating circRNA availability. CircRNA availability is driven by a complex network of factors regulating both their synthesis and clearance from the cell. CircRNA synthesis depends on specific components that promote back splicing, and on the expression of debranching enzymes that regulates the degradation of lariats. On the other hand, circRNA clearance from the cells can occur through different mechanisms of degradation: Ago-2-dependent degradation, m6A-triggered degradation, and GW182 expression. Furthermore, the active secretion of circRNAs through extracellular vesicles (EVs) can be a possible mechanism for circRNA clearance. The availability of specific circRNAs inside the pancreatic β-cells can affect their key functions.

The presence of circular transcripts has long been neglected and considered a rare and poorly relevant event. It has now become clear that this particular class of transcripts is abundant in mammalian cells and constitutes an important fraction of the transcriptome. Recent progress in circRNA research has uncovered new aspects about their biological functions, implicating them both in physiological and pathological processes. However, despite the fact that thousands of circRNAs have already been identified, the field is still in its infancy, and only for very few of them we have information about their involvement in the regulation of cellular activities. The majority of the circRNAs might have a unique, but still unknown function, or act together to accomplish a specific task. However, it is possible that a fraction of the detected circRNAs merely reflects byproducts of the splicing reaction that are expressed at concentrations insufficient to significantly affect cellular functions. CircRNAs have been reported to be abundant in aging cells [91,92]. Given the resistance of circRNAs to exonucleolytic decay, this may reflect the unavoidable accumulation of splicing error byproducts without a biological function. However, even if individually not very abundant, the accumulation of a large number of these splicing byproducts may potentially impact on cellular function and contribute to cellular senescence. A large number of circRNAs is expressed in β-cells, some of which originate from key genes controlling the differentiation and the function of the insulin-secreting cells or from diabetes susceptibility genes. We have initial evidence indicating that at least some of these circular transcripts participate in the regulation of β-cell activities and are dysregulated under diabetes conditions. Future and more systematic studies will most probably uncover the involvement of many additional circRNAs in the control of β-cell functions. A better understanding of how these crucial circRNAs are produced and degraded inside the cells, may potentially favor the design of strategies allowing to positively regulate the activities of the insulin-secreting cells. As previously discussed, circRNA availability is driven by a complex network of factors regulating both their synthesis and clearance from the cell that are still incompletely understood (Figure 4). CircRNA synthesis depends on specific conditions that promote back splicing instead of linear splicing, or that inhibit lariat debranching to favor the generation of lariat-derived circRNAs (see Section 2). On the other hand, clearance of circRNA has been proposed to occur through different cellular pathways (see Section 2) or by active secretion through extracellular vesicles, adding another level of complexity [93,94] (Figure 4). The processes modulating the level of the circRNAs expressed in pancreatic β-cells have not been investigated so far. The elucidation of these regulatory mechanisms will not only help with understanding the causes of circRNA dysregulation under diabetes conditions, but will also set the basis for the development of new tools to modulate/control the functions of these non-coding RNAs in pancreatic β-cells.

The discovery of circRNAs has opened an entirely new investigation field and the study of the role of these non-coding RNAs promises to take a central place in islet research in the coming years. Further validation of the sequencing data will help uncovering new circRNAs involved in different aspects of β-cell biology and will guide the selection and the use of circular transcripts as indicators of β-cell health or demise.

## Data Availability

Not applicable.
